# Effect of Roughage Source on the Composition and Colonization of Rumen Bacteria and Methanogens in Dumont and Mongolian Sheep

**DOI:** 10.3390/ani15142079

**Published:** 2025-07-14

**Authors:** Wenliang Guo, Hongyang Liu, Yue Wang, Meila Na, Ran Zhang, Renhua Na

**Affiliations:** 1 College of Animal Science, Inner Mongolia Agricultural University, Hohhot 010018, China; 18686197338@163.com (W.G.);; 2 Institute of Environment and Sustainable Development in Agriculture, Chinese Academy of Agricultural Sciences, Beijing 100081, China

**Keywords:** roughage, sheep, breed, growth performance, bacteria, methanogens

## Abstract

Understanding the influence of the sheep breed and roughage source on the composition of rumen bacteria and methanogens is essential for optimizing roughage efficiency. The experiment employed a 2 × 2 factorial design. Twenty-four Dumont and Mongolian sheep (initial body weight of 18.94 ± 1.01 kg) were randomly assigned by breed to two dietary treatment groups (AH: alfalfa hay; CS: corn straw); the experiment lasted 90 days. The results showed that sheep fed alfalfa hay diets had a higher feed intake and weight gain, and Dumont sheep had a higher feed intake than Mongolian sheep (*p* < 0.05). The diversity and composition of ruminal bacteria and methanogens differed between Dumont and Mongolian sheep fed either AH or CS diets. The taxonomic analysis revealed a distinct clustering pattern based on the roughage source, but not on the breed. When fed a corn straw diet, the bacterial Chao1 index of Dumont sheep increased (*p* < 0.05), while the diversity and richness of methanogens in Mongolian sheep increased (*p* < 0.05). Additionally, we have identified unique biomarkers for the rumen bacteria and methanogens of Dumont and Mongolian sheep in response to different roughage sources. The results suggest that the differences in the microbiota of the sheep were associated with the roughage source and breed. The higher growth performance of Dumont sheep might be attributed to the increase in bacterial diversity and the decrease in methanogenic bacteria diversity.

## Simple Summary

The rumen microbial community plays a critical role in the production efficiency of ruminants and may be influenced by factors such as the animal breed and diet. We investigated the effects of breed (Dumont and Mongolian sheep) and roughage source (alfalfa hay and corn straw) on the rumen microbial community. We found that the roughage type strongly affects both the feed efficiency and rumen microbial community composition, and this effect was influenced by the interaction between the breed and roughage source. The two sheep breeds fed different roughages exhibited distinct rumen microbial communities, indicating that ruminal microbiota are simultaneously affected by diet and genetic differences, thereby altering the feed efficiency.

## 1. Introduction

Roughage in the diet is crucial for rumen function and growth in fattening sheep. High-quality roughage with low NDF concentrations promotes feed intake and increases weight gain [1]. Although alfalfa hay is a desirable roughage for ruminants, its use increases feed costs per kilogram of mutton [2]. Corn straw, a by-product of corn harvesting, is widely used in Inner Mongolia, China, to address feed shortages [3]. However, the characteristics of the high-fiber and low-protein content in straw restrict its feeding value [4], while also increasing ruminal methane production [5]. Various methods, including chemical [6], physical [7], and biological treatments [2], have been developed to improve the digestibility of straw by rumen, but their application is limited by environmental pollution, high costs, and labor requirements.

Bacteria, which account for about 85% of the microbiota, mainly degrade roughage by secreting enzymes [8]. Methanogens (2–4% of rumen microbiota) utilize bacterial fermentation products to produce methane, resulting in 2–12% of the host’s ingested feed energy loss [9]. Thus, analyzing both bacteria and methanogens can help researchers to understand the degradation and utilization of roughage [10,11]. Several studies have revealed that changing the proportion and source of roughage in the diet and, thereby, the composition of rumen bacteria and methanogens, influences the phenotypic traits and methane production of the host [10,12,13,14].

Apart from diet, host genetics also regulate the rumen microbial community and function [7]. For example, Tibetan sheep exhibit higher rumen bacterial diversity and Firmicutes abundance than Hu sheep, due to their adaptation to high-altitude environments [15,16]. Similarly, the rumen microbial composition and function in Hu sheep and Suffolk ewes are different during pregnancy, which regulates the intestinal immunity level of the lambs [17]. More recently, Xiang et al. [18] studied breed differences in regard to the ruminal bacteria composition, using Dumont and Mongolian sheep that were fed the same diet. They found that the dominant bacterial genera were common to both breeds and that there were also clear breed differences that were species specific, which in turn changed the meat quality traits and muscle fatty acid composition of the hosts. King et al. [19] found that when lactating Jersey and Holstein dairy cows were fed the same diet, the composition of rumen methanogens was significantly different, with greater diversity noted in Holsteins. Similarly, Samantha et al. [10] found that rumen bacteria were impacted by both the breed and diet, whereas methanogens were primarily breed dependent. Furthermore, particular ruminal microbiomes have been found to be more highly clustered within certain breeds [20], which supports the hypothesis that ruminal microbiome symbiotic evolution is dependent on host control [21]. Therefore, rumen microorganisms may be influenced by both the genetics of the host and their diet, which will determine which microbes flourish in a particular rumen environment, thus determining the animal’s productivity [22,23].

Dorper sheep are world-renowned for their rapid growth and lean meat proportion. Mongolian sheep (MS), a Chinese indigenous sheep breed, is known for its strong adaptability and it can maintain good health in harsh environments by adapting well to coarse feed. Dumont sheep (DS) is a new meat sheep breed in China, cultivated by mating Dorper rams (terminal sires) with Chinese Mongolian ewes. Dumont sheep is adapted to desert and semi-desert grasslands and is now the main source of mutton in northern China [24,25]. Existing studies have shown that there are differences in the meat traits and rumen bacteria between Dumont sheep and Mongolian sheep [18]. However, the dominant species of bacteria and methanogens in these sheep when fed with different roughage sources remain unclear. Recent studies have highlighted a reciprocal interplay between rumen microbiota and host genetic variations [26], jointly determining their phenotypic traits that are associated with unique microbial communities [27,28]. In this study, we aimed to determine the impact of different roughage sources on the growth performance and ruminal microbial populations of different sheep breeds. We hypothesized that breed differences would exist, and that the microbial community of each breed would respond differently to a particular type of roughage. We also hypothesized that Dumont sheep efficiently utilize roughage through specific microbiota.

## 2. Materials and Methods

### 2.1. Animal and Experiment Design

The study was conducted from April to July 2024 at the Sheep Experimental Station of Inner Mongolia Agricultural University, located in Tumed Left Banner (40°53′ N and 111°13′ E), Hohhot City, Inner Mongolia, China. Twenty-four sheep (12 Dumont sheep and 12 Mongolian sheep; initial body weight 18.94 ± 1.01 kg, average BCS 2.9, average feed intake 800 g/d) were used in this experiment. A 2 × 2 factorial randomized block design was employed, involving two sheep breeds (Dumont sheep, DS; Mongolian sheep, MS) and two roughage sources (alfalfa hay, AH; corn stalk, CS). Twenty-four sheep were randomly assigned to four treatment groups (*n* = 6 per group): DSAH, DSCS, MSAH, and MSCS. Two isocaloric and isonitrogenous diets were formulated using soybean meal, corn husk, and bran, meeting the NYT816-2021 nutrient requirements [29] (Table 1). The cutting length of the corn stalks and alfalfa hay was 20 mm ± 3 mm, the length of the concentrate pellets was 10 mm, and the diameter was 5 mm. The experiment lasted 105 days, including a 15-day transition period and a 90-day trial period. The sheep were housed individually in outdoor pens (1.0 × 1.0 m^2^), with sand bedding, and fed a total mixed ration (TMR) twice daily (09:00 and 16:00), with ad libitum intake. All the experimental procedures followed the guidelines approved by the Animal Welfare and Ethics Committee of Inner Mongolia Agricultural University (Approval No. NND2024053).

### 2.2. Intake, Growth Performance, and Rumen Sample Collection

The feed intake of the sheep in all four groups was recorded weekly during the 90-day experimental period. On days 30, 60, and 90 of the experiment, the sheep were weighed and rumen fluid samples were collected orally, using a sterile stomach tube, after 16 h of feeding (miniature desktop vacuum pump, GL-802, Jiahang Bochuang Technology Co., Ltd., Beijing, China). A single sample was collected in two fractions: the first 50 mL was discarded to avoid contamination from the sampling tube, and the remaining 150 mL was filtered through four layers of gauze. Then, 2 mL of the filtered fluid was transferred into a sterile cryovial, rapidly frozen in liquid nitrogen, and stored at −80 °C for subsequent analysis of the ruminal bacteria and methanogens.

### 2.3. Rumen Bacterial and Methanogen DNA Extraction and Analysis

Microbial DNA was extracted from 200 mg of each rumen sample using the E.Z.N.A.^®^ soil DNA Kit (Omega Bio-tek, Norcross, GA, USA), following the manufacturer’s protocol. The V4 region of the bacterial 16S gene and methanogen 16S gene were amplified separately, using primer pairs 338F/806R (5′-ACTCCTACGGGAGGCAGCAG-3′/5′-GGACTACHVGGGTWTCTAAT-3′) and MLfF/MLfR (5′-GGTGGTGTMGGATTCACACARTAYGCWACAGC-3′/5′-TTCATTGCRTAGTTWGGRTAGTT-3′), respectively. The PCR reactions (20 µL) contained 4 µL 5 × FastPfu Buffer, 2 µL 2.5 mM dNTPs, 0.4 µL forward primer (5 µM), 0.8 µL reverse primer (5 µM), 0.2 µL BSA, 10 ng template DNA, and ddH_2_O made up to 20 µL. Amplification was performed using a T100 Thermal Cycler (Bio-Rad, Hercules, CA, USA) under the following conditions: 95 °C for 30 s and 60 °C for 30 s (30 cycles) [30]. The PCR products were purified using a Zymoclean Gel DNA Recovery Kit (Zymo Research, Irvine, CA, USA), after electrophoresis in AquaPōr LM agarose. Quantification was performed using a Qubit 4.0 Fluorometer and qPCR (Kapa Biosystems, Wilmington, MA, USA). Paired-end sequencing (2 × 250 bp) was conducted on the Illumina NovaSeq 6000 platform at Majorbio Bio-Pharm Technology Co., Ltd., Shanghai, China. The PCR products were purified using Zymoclean gel extraction in AquaPōr LM low-melt agarose (National Diagnostics, Atlanta, GA, USA), using a Zymoclean Gel DNA Recovery Kit (Zymo Research, Irvine, CA, USA). After, the constructed library was quantified using Qubit and real-time PCR (Kapa Biosystems, Wilmington, MA, USA). Sequencing was performed using the Illumina NovaSeq 6000 sequencing platform (Majorbio Bio-Pharm Technology Co., Ltd., Shanghai, China).

The raw sequences were processed using FASTP (v0.19.6) for quality control and FLASH (v1.2.7) for merging. The operational taxonomic units (OTUs) were clustered at a 97% similarity threshold, using DADA2 in QIIME 2 (v1.8.0). The alpha diversity (Chao1, Shannon) was calculated and compared using the Wilcoxon rank-sum test. The beta diversity was analyzed via PCoA, based on the Bray–Curtis distance, with ANOSIM testing. Differential taxa were identified using LEfSe 1.19.1 (LDA score > 2.5, *p* < 0.05).

### 2.4. Data Processing and Analysis

The growth performance, bacteria, and methanogens data were analyzed using a general linear mixed model in SAS 9.2 (SAS Institute Inc., Cary, NC, USA), with the breed (Dumont vs. Mongolian sheep), roughage type (alfalfa hay vs. corn straw), and their interaction as fixed effects, and individual sheep as the random effect. Model assumptions were verified using the Shapiro–Wilk normality test and Levene’s homogeneity of variance test. For significant effects (*p* < 0.05), post hoc comparisons using Tukey’s HSD test were used when the interaction was significant, otherwise the least squares means were compared, and values were considered significant at *p* < 0.05 and extremely significant at *p* < 0.01. The data were presented as averages. The bacteria relative abundance, methanogen relative abundance, and the relationship between the bacteria and methanogens in relation to growth performance were assessed using Spearman’s rank correlation, with coefficients >|0.4|, *p* < 0.05 considered significant.

## 3. Results

### 3.1. Growth Performance

The effects of the roughage type on the growth performance of Dumont crossbred sheep and Mongolian sheep are presented in Table 2. No significant differences in the weight gain were present in the sheep among the treatment groups at 30 and 60 days. By 90 days, the sheep fed AH diets showed greater weight gain than those fed CS diets (*p* < 0.05). A similar pattern was observed for feed intake, with higher values in the AH groups compared to the CS groups (*p* < 0.05), and Dumont sheep had higher values than that of the Mongolian sheep. There were no differences between the treatments in regard to feed efficiency.

### 3.2. Bacterial Community Composition

**Figure 1:**
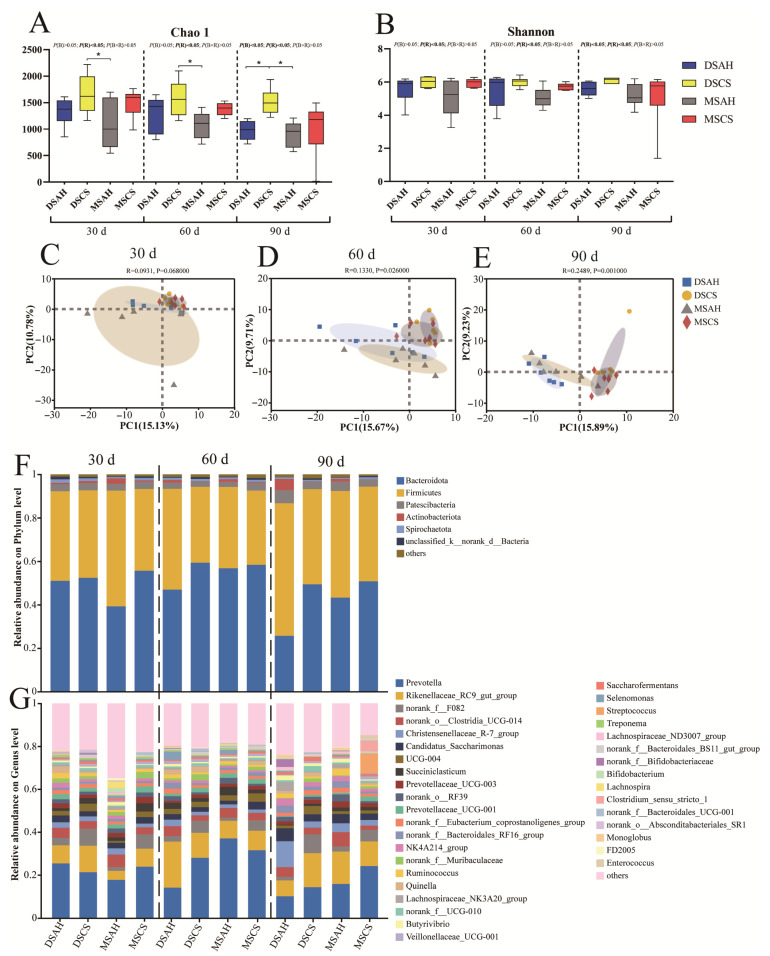
Dietary roughage sources altered rumen bacterial diversity and composition in Dumont sheep and Mongolian sheep. (**A**,**B**) Chao1 and Shannon index of alpha diversity under ASV level at 30, 60, and 90 days of the experiment. (**C**–**E**) Principal analysis (PCA) under ASV level at 30, 60, and 90 days of the experiment, different shades of color represent different treatment groups. (**F**,**G**) Bacteria taxa averaged at the phylum and genera level at 30, 60, and 90 days of the experiment. DSAH: Dumont sheep fed alfalfa hay, DSCS: Dumont sheep fed corn stalk, MSAH: Mongolian sheep fed alfalfa hay, MSCS: Mongolian sheep fed corn stalk, *n* = 6. “*” indicates a significant difference and “*p* < 0.05”.

A total of 4,395,941 reads (1,839,388,682 sequences) were obtained after quality filtering and clustered into 50,253 amplicon sequence variants (ASVs) per sample. For the alpha diversity analysis, the CS diets’ Chao1 index was greater than the AH diets’ at 30, 60, and 90 days (*p* < 0.05) (Figure 1A). Additionally, Dumont sheep had a higher Chao1 index than that of the Mongolian sheep (*p* < 0.05), but no difference was observed in the Shannon index among the four treatment groups (Figure 1B). The PCA score map showed that the separation distance between the bacterial communities in the AH and CS groups gradually widened as the experiment progressed, but the impact of the sheep breed resulted in mostly overlapping distributions in regard to the different experimental dates (Figure 1C–E).

Moreover, seven bacterial phyla with a relative abundance >1% were detected across all the individuals, with *Firmicutes* and *Bacteroidetes* being the most dominant (Figure 1F,G). At the genus level, 36 exceeded 1% abundance, with the predominant genera being *Prevotella*, *Rikenellaceae_RC9_gut_group*, *norank_f__F082*, *norank_o__Clostridia_UCG-014*, and *unclassified_f__Lachnospiraceae*. Using MetaStat analysis, we compared the rumen bacterial composition among the four treatment groups at 30, 60, and 90 days of the experiment. The relative abundance of *Prevotella* was higher in the Mongolian sheep than in the DSAH group at 60 days (*p* < 0.01). The relative abundance of *Rikenellaceae_RC9_gut_group* was higher in the Dumont sheep than in the Mongolian sheep and greater for the CS diets relative to the AH diets at 60 days (*p* < 0.01). The relative abundance of *norank_f__F082* was higher in the CS than in the AH group at 30, 60, and 90 days (*p* < 0.01). In addition, at 90 days of the experiment, the relative abundance of *unclassified_f__Lachnospiraceae* was higher in the Dumont sheep than in the Mongolian sheep, the relative abundance of *Christensenellaceae_R-7_group* was higher in the Dumont sheep than in the Mongolian sheep and greater for the AH diets relative to the CS diets, and the relative abundance of *UCG-004* was higher in the CS than in the AH group.

**Figure 2:**
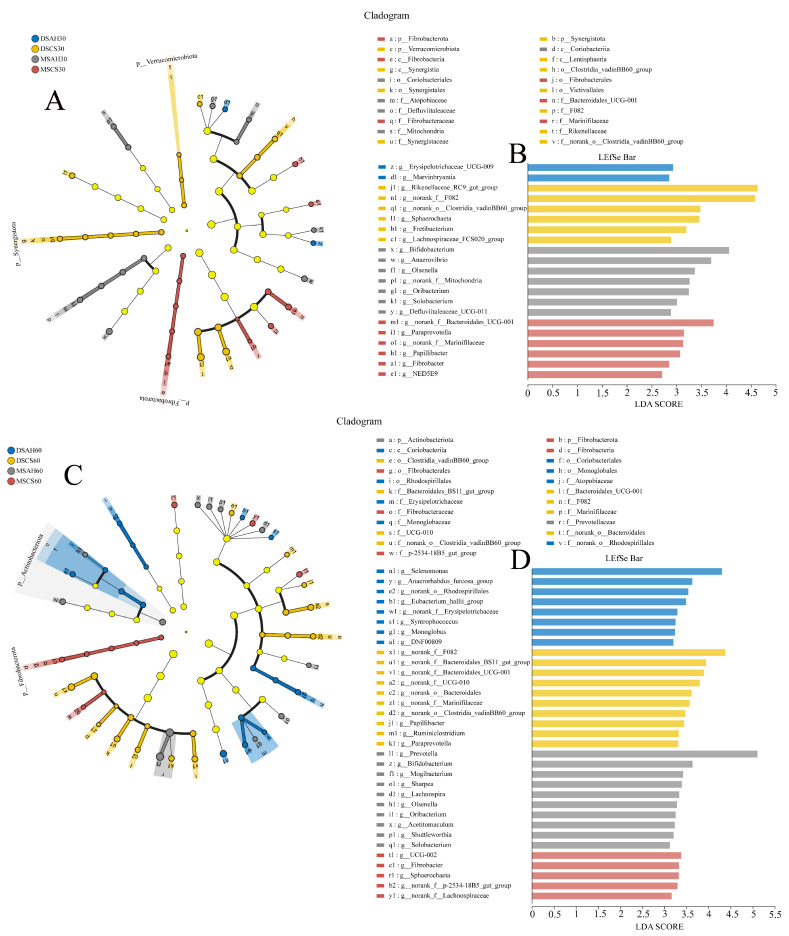
The rumen bacterial biomarkers (genera level) with a linear discriminant analysis (LDA) score > 2.5, using LEfSe analysis. (**A**,**B**) A cladogram and LEfSe bar chart at 30 days of the experiment, a-c1 represents distinct bacterial taxonomic units. (**C**,**D**) A cladogram and LEfSe bar chart at 60 days of the experiment. Different colors indicate different treatments, a–y1 represents distinct bacterial taxonomic units. The colored dots from inner to outer levels represent phylum, class, order, family, and genus levels. DSAH: Dumont sheep fed alfalfa hay, DSCS: Dumont sheep fed corn stalk, MSAH: Mongolian sheep fed alfalfa hay, MSCS: Mongolian sheep fed corn stalk, *n* = 6.

**Figure 3:**
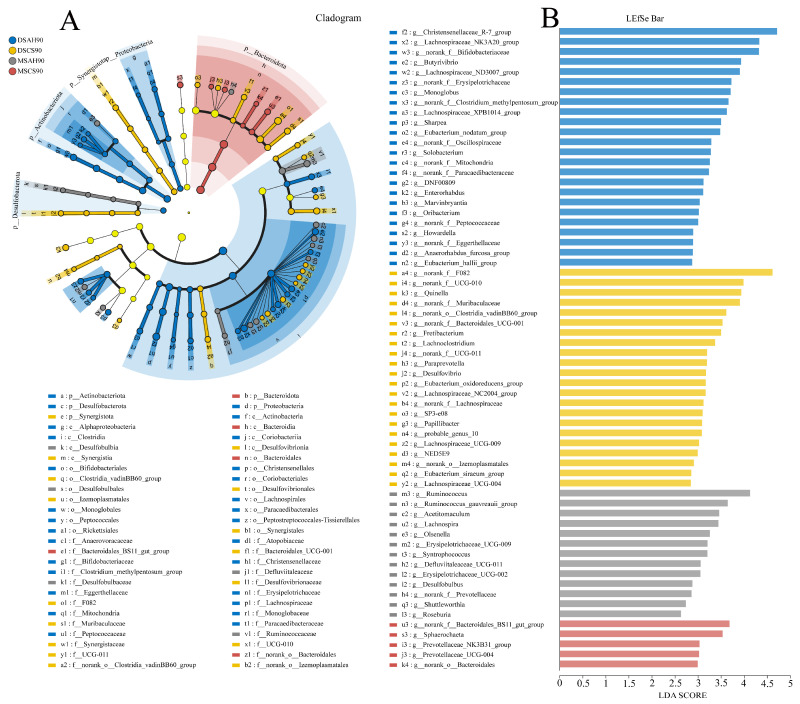
The rumen bacterial biomarkers (genera level) with a linear discriminant analysis (LDA) score > 2.5, using LEfSe analysis. (**A**,**B**) A cladogram and LEfSe bar chart at 90 days of the experiment, a-k4 represents distinct bacterial taxonomic units. DSCS: Dumont sheep fed corn stalk, MSAH: Mongolian sheep fed alfalfa hay, MSCS: Mongolian sheep fed corn stalk, *n* = 6.

To further investigate the phylogenetic relationships among the species at the genus level, representative sequences of the genera were obtained through multiple sequence alignment (MSA). An evolutionary tree was then constructed to visualize the relationships at the genus level. LEfSe analysis (with a logarithmic LDA score threshold of 2.5) was applied to identify species-specific biomarkers for each group across different experimental time points (Figure 2 and Figure 3). As the experiment progressed, the number of ruminal microbiomes specific to the different breeds gradually increased, and further differentiation occurred when the sheep were fed different types of roughage, which was particularly notable in the Dumont sheep. On the 30th day of the experiment, the DSAH, DSCS, MSAH, and MSCS groups exhibited two, six, seven, and six biomarker bacterial genera, respectively. On the 60th day, the same groups exhibited eight, ten, ten, and five biomarker bacterial genera, respectively. On the 90th day, the unique bacterial phylum in the DSAH group were Desulfobacterota, Actinobacteriota, and Proteobacteria, which contained 24 bacterial genera, such as *Christensenellaceae_R-7_group*, *unclassified_f__Lachnospiraceae*, and *Bifidobacteriaceae*. The bacterial phylum, Synergistotap, and 22 bacterial genera, such as *norank_f__F082*, *UCG-010*, and *Quinella*, were identified as biomarkers in the DMCS group. The unique bacteria genera in the MSAH group involved 13 genera, including *Ruminococcus*, *Acetitomaculum*, and *Lachnospira*. In addition, the bacterial phylum, Bacteroidota, and five biomarker bacterial genera, such as *Bacteroidales_BS11_gut_group*, *Shuttleworthia*, and *Roseburia* were enriched in the MSCS group.

### 3.3. Ruminal Bacterial Communities

**Figure 4:**
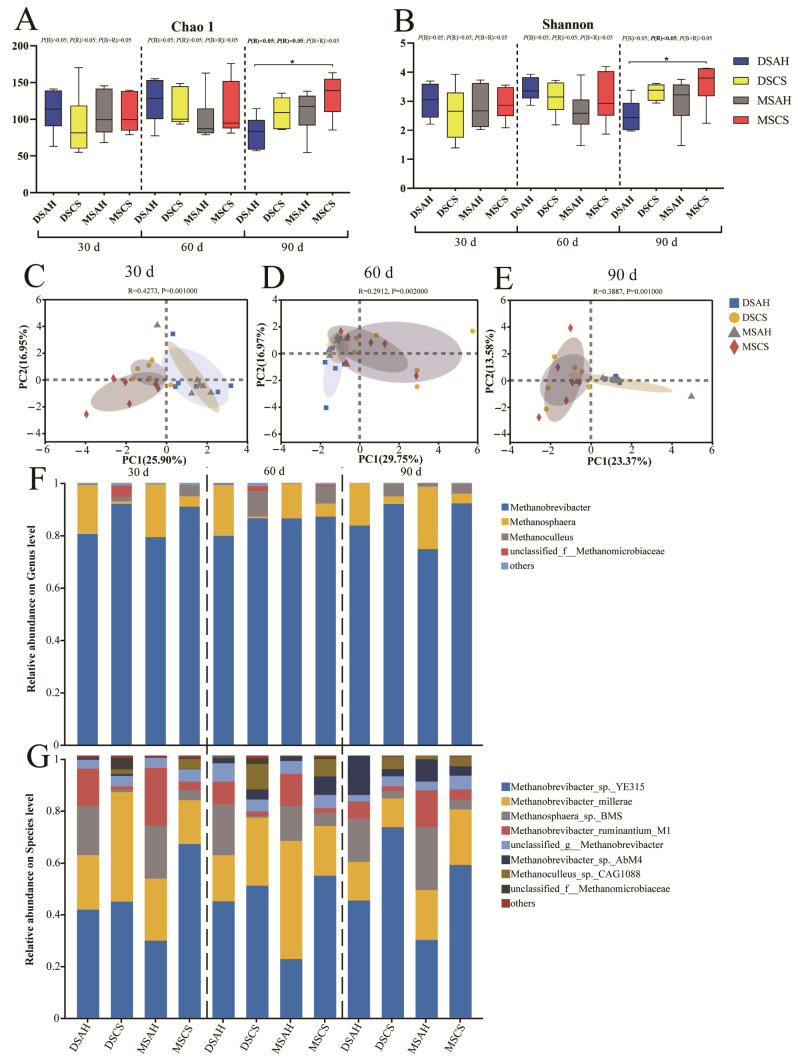
Dietary roughage sources altered rumen methanogen diversity and composition in Dumont sheep and Mongolian sheep. (**A**,**B**) Chao1 and Shannon index of alpha diversity under ASV level at 30, 60, and 90 days of the experiment. (**C**–**E**) Principal analysis (PCA) under ASV level at 30, 60, and 90 days of the experiment, different shades of color represent different treatment groups. (**F**,**G**) Methanogen taxa averaged according to genera and species level at 30, 60, and 90 days of the experiment. DSAH: Dumont sheep fed alfalfa hay, DSCS: Dumont sheep fed corn stalk, MSAH: Mongolian sheep fed alfalfa hay, MSCS: Mongolian sheep fed corn stalk, *n* = 6. “*” indicates a significant difference and “*p* < 0.05”.

A total of 2,377,819 reads (985,942,217 sequences) were obtained after quality filtering and clustered into 3928 ASVs per sample. In regard to the alpha diversity analysis, the Chao1 index was greater in the Mongolian sheep than the Dumont sheep and greater for the CS diets relative to the AH diets at 90 days (*p* < 0.05) (Figure 4A). Additionally, the Dumont sheep had a higher Shannon index than that of the Mongolian sheep at 90 days (*p* < 0.05) (Figure 4B). The PCA score map shows the separation distance between the methanogen communities in the AH and CS groups at 30, 60, and 90 days; the impact of the sheep breed resulted in mostly overlapping distributions in regard to the different experimental dates (Figure 4C–E).

**Figure 5:**
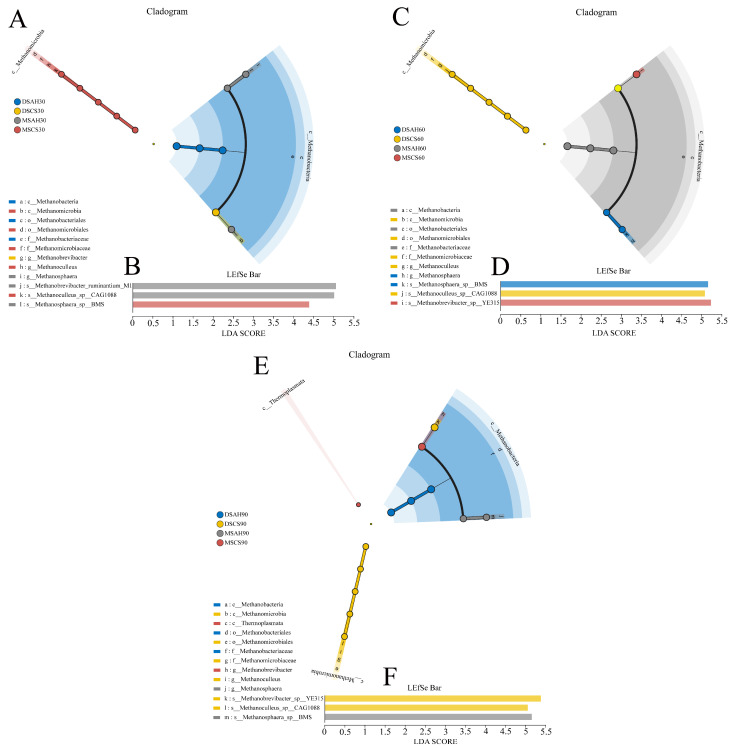
The rumen bacterial biomarkers (genera level) with a linear discriminant analysis (LDA) score > 2.0, using LEfSe analysis. (**A**,**B**) A cladogram and LEfSe bar chart at 30 days of the experiment. (**C**,**D**) A cladogram and LEfSe bar chart at 60 days of the experiment. (**E**,**F**) A cladogram and LEfSe bar chart at 90 days of the experiment, a–m represents distinct bacterial taxonomic units. DSAH: Dumont sheep fed alfalfa hay, DSCS: Dumont sheep fed corn stalk, MSAH: Mongolian sheep fed alfalfa hay, MSCS: Mongolian sheep fed corn stalk, *n* = 6.

Ninety-nine percent of the methanogen were identified as belonging to the archaea phylum, with the predominant genera being *Methanobrevibacter*, *Methanosphaera*, and *Methanoculleus*. Using MetaStat analysis, we compared the composition of rumen methanogens among the four treatment groups at 30, 60, and 90 days of the experiment (Figure 5). On day 30, the relative abundance of *Methanobrevibacter* was higher, while that of *Methanosphaera* was lower in the CS group compared to the AH group. This trend persisted at day 60 and 90, with *Methanosphaera* remaining at lower levels and *Methanoculleus* exhibiting significantly higher abundance (*p* < 0.01) in the CS group relative to the AH group. At the species level, eight species exceeded 1% abundance. The predominant species were *Methanobrevibacter_*sp.*_YE315*, *Methanobrevibacter_millerae*, *Methanosphaera_*sp.*_BMS*, *Methanobrevibacter_ruminantium_M1*, and *unclassified_g__Methanobrevibacter*. We used LEfSe analyses to identify the methanogen biomarkers for the four treatment groups on different experimental dates. On day 30, there was no enrichment of methanogen in the DS groups, *Methanobrevibacter_ruminantium_M1* and *Methanoculleus_*sp.*_CAG1088* were enriched in the MSAH group, while *Methanosphaera_*sp.*_BMS* was more abundant in the MSCS group. On day 60, *Methanosphaera_*sp.*_BMS* was enriched in the DSAH group, *Methanoculleus_*sp.*_CAG1088* was more abundant in the DSCS group, and *Methanobrevibacter_*sp.*_YE315* was more abundant in the MSCS group. On day 90, *Methanobrevibacter_*sp.*_YE315* and *Methanoculleus_*sp.*_CAG1088* were enriched in the DSCS group, while *Methanosphaera_sp._BMS* was more abundant in the DSAH group. These results reveal that the anti-methanogenic effect observed between the AH and CS diets was initially more pronounced in Dumont sheep compared to Mongolian sheep during the early experimental phase, and the influence of the roughage source gradually expanded throughout the 90-day experimental period.

### 3.4. Ruminal Metabolomics Analysis

**Figure 6:**
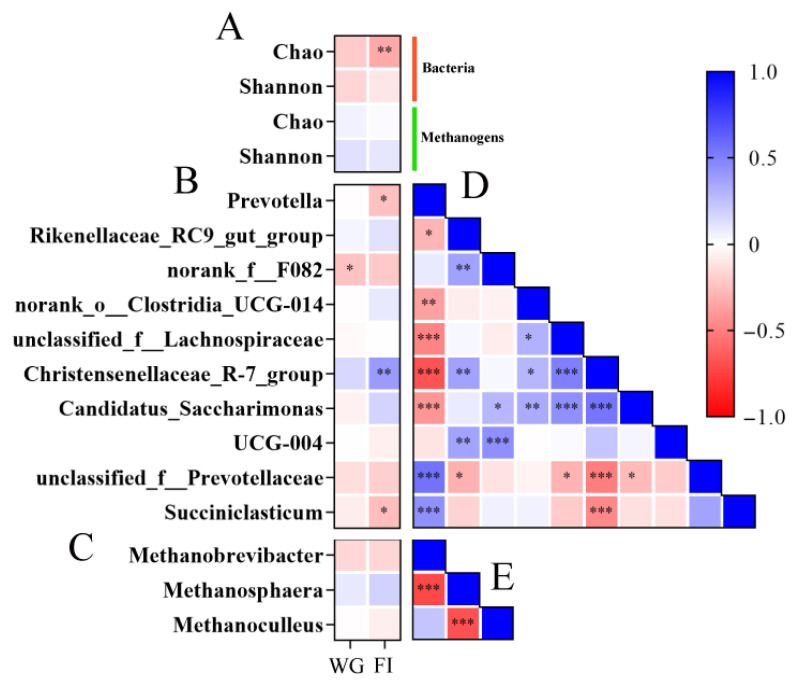
The relationship between growth performance, bacteria, and methanogen genera (top 10). (**A**) Heat map of bacteria and methanogen alpha diversity index to growth performance. (**B**) Heat map of bacteria genus level in terms of relative abundance to growth performance. (**C**) Heat map of methanogen genus level in terms of relative abundance to growth performance. (**D**) Triangular heatmap of bacteria. (**E**) Triangular heatmap of methanogens. “*”, “**”, and “***” indicates a significant difference and “*p* < 0.05”, “*p* < 0.01”, and “*p* < 0.001”, respectively. WG: weight gain; FI: feed intake.

Heatmap correlation analysis was employed to determine the correlations between the relative abundance of the microorganisms (top 10 bacterial genera and methanogens) and growth performance. The weight gain displayed a negative correlation with the relative abundance of *norank_f__F082* (*p* < 0.05). The feed intake displayed negative correlations with the bacterial Chao1 index (*p* < 0.01), *Prevotella* (*p* < 0.05), and *Succuriclasticum* (*p* < 0.05), while positively correlating with *Christensenellaceae_R-7_group* (*p* < 0.01) (Figure 6A–C).

The correlations among the bacteria at the genus level were analyzed using triangular heatmaps. The results indicated that the relative abundances of *Prevotella*, *unclassified_f__ Prevotellaceae*, and *succiniclasticum* were positively correlated with each other (*p* < 0.01). In contrast, they were negatively correlated with *Rikenellaceae_RC9_gut_group*, *norank_o__Clostridia_UCG-014*, *unclassified_f__Lachnospiraceae*, *Christensenellaceae_R-7_group*, and *Candidatus_Saccharimonas* (*p* < 0.01). The relative abundance of *Rikenellaceae_RC9_gut_group* was positively correlated with *norank_f__F082*, *Christensenellaceae_R-7_group*, and *UCG-004* (*p* < 0.01). Additionally, *norank_o__Clostridia_UCG-014*, *unclassified_f__Lachnospiraceae*, *Christensenellaceae_R-7_group*, and *Candidatus_Saccharimonas* demonstrated positive correlations among themselves (*p* < 0.01). At the methanogen genus level, the relative abundance of Methanobrevibacter was negatively correlated with Methanosphaera (*p* < 0.01) (Figure 6D,E).

## 4. Discussion

Due to their low cost, corn stalks are commonly mixed with soybean meal in diets for fattening lambs. However, its complex fiber structure leads to poor palatability and a low rumen degradation rate, thereby reducing the production performance of animals [1,2,4,31]. In the present study, although the feed intake of the sheep fed alfalfa hay diets was higher than that of sheep fed corn stalk diets at 30 d and 60 d of the experiment, the weight gain of both breeds did not differ. This result could be explained by the fact that both the alfalfa hay diet and corn straw diet were isoenergy and isonitrogenous. Mater et al. [32] observed that a straw diet was more effective in reducing feed intake than alfalfa hay for sheep during 90-day experimental periods, but there was no difference in growth performance, and the diet was not formulated to be isoenergy and isonitrogenous. In our study, after 90 days of the experiment, the sheep fed alfalfa hay had a higher feed intake and BW compared to those fed corn straw. Similar to our result, Kyawt et al. [2] reported a lower feed intake, body weight gain, and feed efficiency when sheep were fed isonitrogenous diets containing corn straw compared with alfalfa hay. When fed corn straw with high contents of NDF and ADF, the rumen filling effect and insufficient energy supply will lead, as in this case, to a decline in growth performance. Previous studies have reported that the ADF and NDF in rumen dietary contents shows a significant positive correlation with the BW of sheep [33]. Interestingly, regardless of the roughage source, the feed intake of Dumont sheep was always higher than that of Mongolian sheep. This indicates that Dumont sheep have better growth performance, and the influence of the roughage source and breed increase as the experiment progresses.

Rumen bacteria digest feed to produce nutrients for the host, and their population structure is significantly influenced by the diet, which is further differentiated based on the host breed [34]. The diversity index was higher in the DSCS group than in the MSAH group, which indicates that the Dumont sheep fed with corn stalk had a more complicated rumen bacterial network. Costa et al. [35] found that diets with different types of roughage (barley straw, oats haylage, and vetch haylage) significantly altered the ruminal microbial community of Holstein bulls, and feeding animals with oats and vetch haylage raised the ruminal microbial alpha biodiversity. The Bray–Curtis dissimilarities in regard to the principal component analysis indicated that the roughage source had a more decisive influence on the rumen bacteria diversity as the experiment progressed. This might also be related to the age of the host and their dietary preferences [36]. At the phylum level, the bacterial composition is still dominated by *Bacteroidetes* and *Firmicutes*. Increasing the content of ADF and NDF in the diet increased the diversity of rumen microorganisms and the abundance of *Firmicutes* [37]. *Firmicutes* is the main fiber-degrading phylum in rumen. Our study also found that at 90 d of the experiment, the relative abundance of *Firmicutes* in the sheep fed with a corn straw diet was higher than that in the sheep fed with alfalfa hay. At the genus level, *Prevotella* is the predominant genus within *Bacteroidetes*. It secretes diverse digestive enzymes for carbohydrate utilization. The increasing relative abundance of *Prevotella* in Mongolian sheep indicates that the carbohydrate metabolism was improved. Gao et al. [4] reported that the straw diet increased the relative abundance of *Prevotella* and improved the utilization rate of lignin. In contrast, the relative abundance of *Rikenellaceae_RC9_gut_group*, *Lachnospiraceae*, and *Christensenellaceae_R-7_group* increased in the Mongolian sheep. These bacteria are associated with carbohydrate degradation and protein fermentation [38,39], and are positively correlated with daily weight gain [40]. Based on the changes in the relative abundance of these bacteria in different sheep breeds, this suggests that different breeds of sheep have different dominant bacteria for utilizing carbohydrates. Furthermore, it is worth noting that the relative abundance of *norank_f__F082* increased significantly in the rumen of sheep fed a corn straw diet. It was reported by [41,42] that *norank_f__F082* is positively and negatively correlated with the production of propionate and methane, respectively. However, Long et al. [43] also reported that *norank_f__F082* was negatively correlated with the growth performance of goats, which is consistent with our results. In addition, the results of our LEfSe analysis further illustrate that particular ruminal microbiomes are linked to different breeds, which suggests that the bacteria are influenced both by the breed and roughage source. One bacterium is considered to have multiple functions, and simple bacteria complexion could improve rumen energy efficiency [44]. Similarly, Wang et al. [45] found a negative association between weight gain and a low richness of bacteria in goat and sheep. However, the bacterial community composition and function are influenced by multiple factors (diet, roughage quality, breed, age, and time of day) [46]. In this study, the Mongolian sheep were found to have relatively few bacteria biomarkers, and this is more obvious after being fed a straw diet, which may lead to a reduction in rumen microbial fermentation functions. Interestingly, *norank_f__F082* was enriched in the DSCS group, and is known to have the ability to anti-methane and propionate fermentation, which may explain part of the reason for the higher growth performance of the Dumont sheep fed corn straw.

Methanogens produce CH_4_ by regulating the partial pressure of H_2_, causing host energy loss and the greenhouse effect, while also promoting rumen fermentation, whose relative abundance and composition are mainly affected by the cellulose content of the diet [47]. Franzolin et al. [48] found that, compared with Water Buffaloes fed sugar cane and grazing on brizantha, the diversity of methanogens in cattle fed corn silage was relatively high. In addition to dietary factors, the ruminant breed had a significant effect on rumen methanogens in terms of both their abundance and composition. Zhang et al. [49] suggested that Angus, Charolais, and Kinsella cattle harbor different rumen microbiota at both the abundance and activity level. Noel et al. [10] also reported that the rumen microbial community of Holstein and Jersey cows had different levels of response to a dietary intervention, based on 16S rRNA gene sequencing analysis. However, the effect of the Dumont and Mongolian sheep breed on rumen methanogens is not well-known. In the present study, similar to the bacteria results, the Bray–Curtis dissimilarities in regard to the principal component analysis indicated that the methanogen community was influenced by the roughage source, and to a lesser extent by the breed. The MetaStat analysis also indicated that *Methanobrevibacter* and *Methanoculleus* were enriched in the corn straw diet, while *Methanosphaera* was enriched in the alfalfa diet. However, there was no significant difference in the methanogen community structure among the different breeds. *Methanobrevibacter* and *Methanosphaera* belong to the *Methanobacteria* class, which usually accounts for more than 90% of methanogen 16S rRNA gene reads. *Methanoculleus* belongs to the Methanomicrobia class. *Methanobrevibacter* and *Methanoculleus* perform methanogenesis from CO_2_ with H_2_ and formate, while *Methanosphaera* uses H_2_ and methanol for methanogenesis [50]. This also verifies the anti-methane effect of alfalfa diets [9]. At 90 days into the experiment, as we originally hypothesized, the diversity and abundance of methanogens in the DSAH group were both lower than that of the MSCS group. This suggests that the two sheep breeds had different levels of response to the dietary roughage source, which is in line with other studies [10]. The LEfSe analysis found that no methanogen species were enriched in the Dumont sheep on the 30th day of the experiment. As the experiment progressed, the enrichment of methanogen species in the different groups may be due to differences in the rumen fermentation pathway [51,52], but reports on the functions of these methanogen species are still lacking.

The growth performance of the host was strongly correlated with rumen bacteria in our study. The bacterial Chao1 index was negatively correlated with feed intake, which also indicates that simple bacteria community composition determines the rumen fermentation capacity, potentially influencing the growth performance of the host [50]. Similar to the results in our study, Long et al. [42] found a negative correlation between *norank_f__F082* and growth performance in goats. *Prevotella* was positively correlated with *unclassified_f__ Prevotellaceae* and *succiniclasticum*, and negatively correlated with feed intake and other bacterial genera. Although there are reports pointing out that the relative abundance of *Prevotella* and *Succiniclasticum* was positively correlated with protein metabolism and carbohydrate metabolism [53], it was negatively correlated with feed intake in this study. We speculate that *Prevotella* and *Succiniclasticum* may be dominant genera when the feed intake is low, hindering the reproduction of other bacteria in rumen. *Christensenellaceae_R-7_group*, *Rikenellaceae_RC9_gut_group*, and *Lachnospiraceae* have been found to naturally secrete enzymes that can degrade cellulose to promote the decomposition of fiber in rumen [54] and, thereby, enhance growth performance [40]. In addition, our findings revealed that *Methanobrevibacter* was negatively correlated with *Methanosphaera*, which is due to the fact that different substrates are produced after the fermentation of straw or alfalfa [55]. These results indicate that there is always a certain pattern of association among bacteria, methanogens, and growth performance, despite perturbations in dietary roughage. However, this relationship becomes more variable under the control of different feeding preferences and physiological metabolism of the host. It has been speculated that host genetics could influence several biological functions in animals, for instance, eating frequency, feed intake, rumen size, and rumen passage rate [56]. In our study, the feed intake of the Dumont sheep was higher than the other breed, which would further increase the volume of the rumen and stimulate the growth of papillae, thereby resulting in the absorption of more nutrients [13]. Furthermore, the rumen passage rate and movement patterns of different breeds also affect the population and absorption of rumen microorganisms [53,57]. The correlation among host genetics, rumen microorganisms, and diet remains an area in need of further investigation. Our findings provide insights into the fact that the response of the microbiome to diet is controlled through host genetic selection.

## 5. Conclusions

In the present study, we found that as the experiment progressed, both the breed and roughage source had an influence on the growth performance and the rumen bacteria and methanogen community composition of sheep. The roughage had a greater effect on the rumen bacteria and methanogen community composition, and the two sheep breeds showed different levels of response to the different roughage sources. When fed a corn straw diet, the Dumont sheep showed high bacterial diversity in the rumen, and the Mongolian sheep displayed an increase in methanogen diversity and richness. It is possible that the differences in rumen microbial communities between the two breeds contribute to their distinct growth performance. In addition, we have identified unique biomarkers for the rumen bacteria and methanogens of Dumont sheep and Mongolian sheep in response to different roughage sources. These results provide valuable insights into the rumen microbial adaptation to different roughages. Future studies should be based on a larger sheep group and leverage genetic markers and microbial insights to improve sheep breeding and enhance feed efficiency.

## Figures and Tables

**Table 1:** Ingredient composition and nutritional value of experimental feed (dry matter basis).

Ingredients	Treatment ^1^		Nutrient	Treatment	
	AH	CS		AH	CS
Corn stalk	0	50	Dry matter	87.46	87.40
Alfalfa hay	50	0	Digestible energy, MJ/kg	12.03	12.05
Corn	28.2	26.5	Crude protein	13.23	13.13
Rapeseed meal	0	5	Starch	20.35	20.47
Soybean meal	0	15.1	Acid detergent fiber	20.79	27.82
Corn husk	12.5	0	Neutral detergent fiber	35.61	45.13
Wheat bran	6	0	Ether extract	3.68	3.67
Soybean oil	0.3	0.4	Feed cost, USD/t	450	290
Salt	0.5	0.5	Rumen degradable protein	6.4	5.2
Premix ^2^	2.5	2.5			

^1^ AH: alfalfa hay diet, CS: corn stalk diet. ^2^ The premix contained/kg diet: vitamin A 6000 IU, vitamin D3 2000 IU, vitamin E 15 IU, vitamin K3 1.8 mg, vitamin B1 0.35 mg, vitamin B2 8.5 mg, vitamin B6 0.9 mg, vitamin B12 0.03 mg, D-pantothenic acid 16 mg, nicotinic acid 22 mg, folic acid 1.5 mg, biotin 0.15 mg, Cu 8 mg, Fe 40 mg, Mn 20 mg, Zn 40 mg, I 0.8 mg, Se 0.3 mg, and Co 0.3 mg.

**Table 2:** Effect of roughage source on the growth performance of Dumont and Mongolian sheep.

Items	Treatments ^1^				SEM	*p*-Value ^2^			
	Dumont Sheep		Mongolian Sheep			T	B	R	B × R
	AH	CS	AH	CS					
Weight gain, kg									
30 d	2.30	2.01	2.12	1.89	0.24	0.673	0.548	0.292	0.896
60 d	5.30	4.95	5.02	4.04	0.58	0.466	0.314	0.268	0.594
90 d	5.89 ^a^	4.86 ^ab^	5.23 ^a^	3.92 ^b^	0.49	0.026	0.072	0.012	0.746
Feed intake, kg									
30 d	24.66 ^a^	23.59 ^a^	24.35 ^a^	21.88 ^b^	0.57	0.002	0.016	0.002	0.080
60 d	35.53 ^a^	34.59 ^a^	34.65 ^a^	29.20 ^b^	1.46	0.002	0.010	0.009	0.053
90 d	40.45 ^a^	37.29 ^b^	39.36 ^ab^	32.58 ^c^	1.49	0.001	0.003	0.001	0.049
Feed efficiency									
30 d	0.09	0.08	0.09	0.09	0.01	0.917	0.798	0.551	0.798
60 d	0.15	0.14	0.14	0.14	0.02	0.927	0.641	0.652	0.926
90 d	0.15	0.13	0.13	0.12	0.01	0.419	0.311	0.196	0.769

^1^ AH: alfalfa hay diet, CS: corn stalk diet. ^2^ T = different treatments; B = breed (Dumont sheep × Mongolian sheep); R = roughage (AH × CS); B × R = breed × roughage, breed by roughage interaction. ^a,b,c^ Significant differences within a row with different superscripts (*p* < 0.05), *n* = 6.

## Data Availability

The data presented in this study are available upon request from the corresponding author.

## Abbreviations

AH	Alfalfa hay
CS	Corn stalk
DSAH	Dumont sheep and alfalfa hay diet group
DSCS	Dumont sheep and Corn stalk diet group
MSAH	Mongolian sheep and alfalfa hay diet group
MSCS	Mongolian sheep and Corn stalk diet group
DS	Dumont sheep
MS	Mongolian sheep
